# A small indel mutation in an anthocyanin transporter causes variegated colouration of peach flowers

**DOI:** 10.1093/jxb/erv419

**Published:** 2015-09-10

**Authors:** Jun Cheng, Liao Liao, Hui Zhou, Chao Gu, Lu Wang, Yuepeng Han

**Affiliations:** ^1^Key Laboratory of Plant Germplasm Enhancement and Specialty Agriculture, Wuhan Botanical Garden of the Chinese Academy of Sciences, Wuhan, 430074, P.R.China; ^2^Graduate University of Chinese Academy of Sciences, 19A Yuquanlu, Beijing, 100049, P.R.China

**Keywords:** Anthocyanin, chimera, GST, indel, peach, variegation.

## Abstract

The ornamental peach cultivar ‘Hongbaihuatao (HBH)’ can simultaneously bear pink, red, and variegated flowers on a single tree. Anthocyanin content in pink flowers is extremely low, being only 10% that of a red flower. Surprisingly, the expression of anthocyanin structural and potential regulatory genes in white flowers was not significantly lower than that in both pink and red flowers. However, proteomic analysis revealed a GST encoded by a gene—regulator involved in anthocyanin transport (*Riant*)—which is expressed in the red flower, but almost undetectable in the variegated flower. The *Riant* gene contains an insertion-deletion (indel) polymorphism in exon 3. In white flowers, the *Riant* gene is interrupted by a 2-bp insertion in the last exon, which causes a frameshift and a premature stop codon. In contrast, both pink and red flowers that arise from bud sports are heterozygous for the *Riant* locus, with one functional allele due to the 2-bp deletion or a novel 1-bp insertion. Southern blot analysis indicated that the *Riant* gene occurs in a single copy in the peach genome and it is not interrupted by a transposon. The function of the *Riant* gene was confirmed by its ectopic expression in the *Arabidopsis tt19* mutant, where it complements the anthocyanin phenotype, but not the proanthocyanidin pigmentation in seed coat. Collectively,these results indicate that a small indel mutation in the *Riant* gene, which is not the result of a transposon insertion or excision, causes variegated colouration of peach flowers.

## Highlight

An anthocyanin transporter gene that contains a high frequency of non-transposable element-related mutations is responsible for variegated colouration of flowers in peach.

## Introduction

Colouration is an important agronomic trait that contributes to the ornamental value of plants, which is determined by three major classes of plant pigments: anthocyanin, chlorophyll, and carotenoid. In flowers, anthocyanin is the major pigment and confers red, violet, or blue colours (Tanaka *et al.*, 2008). The anthocyanin biosynthetic pathway has been well studied in a variety of plants (Winkel-Shirley, 2001; Grotewold, 2006). This pathway starts with a condensation of malonyl-CoA and 4-coumaroyl CoA, and is catalysed by multiple enzymes including chalcone synthase (CHS), chalcone isomerase (CHI), flavanone 3-hydroxylase (F3H), flavonoid 3′-hydroxylase (F3′H), flavonoid 3′5′-hydroxylase (F3′5′H), dihydroflavonol 4-reductase (DFR), and UDPG-flavonoid glucosyltransferase (UFGT), to generate anthocyanins. Subsequently, water-soluble anthocyanin is transported into the vacuole.

Two mechanisms have been proposed for anthocyanin transport from the site of synthesis to the vacuole: vesicle-mediated transport and transporter-mediated transport (Grotewold and Davis, 2008; Zhao and Dixon, 2010). The evidence for the former mechanism comes from microscopy observations that anthocyanin accumulates initially in vesicle-like structures alongside the tonoplast that merge with the central vacuole (Zhang *et al.*, 2006; Poustka *et al.*, 2007; Conn *et al.*, 2010). The other mechanism is supported by transporter proteins located in the tonoplast, including multidrug resistance-associated proteins (MRP) and multidrug and toxic compound extrusion (MATE) transporters, shown to mediate anthocyanin transport (Debeaujon *et al.*, 2001; Goodman *et al.*, 2004; Grotewold, 2004; Gomez *et al.*, 2009; Zhao and Dixon, 2009; Francisco *et al.*, 2013). In addition, GSTs also have an essential role in transport of anthocyanins from the ER to the vacuole (Larsen *et al.*, 2003; Conn *et al.*, 2008; Gomez *et al.*, 2011). Initially, GST was proposed to conjugate anthocyanins with glutathione to form stable water-soluble conjugates, which were then transported into vacuoles by ABC transmembrane transporters (Marrs *et al.*, 1995). However, no anthocyanin–glutathione conjugates have been found *in vivo*. Instead, evidence suggests that GST functions as an anthocyanin carrier that may escort anthocyanins from the ER to the tonoplast (Mueller *et al.*, 2000; Sun *et al.*, 2012).

Variegation (variable colouration with patches of different colours) is one of the quality parameters sought after by the plant ornamental industry. Variegation is a common phenomenon, with the mechanism of variegation being studied in numerous plant species. In maize, insertion and excision of transposable elements located in the promoter region of the bronze gene encoding GST causes unstable pigmentation in kernels (Schiefelbein *et al.*, 1988). Transposable elements are also responsible for the unstable pigmentation phenotype in ornamental plants such as petunia (Quattrocchio *et al.*, 1999; Spelt *et al.*, 2000), snapdragon (Noda *et al.*, 1994), carnation (Itoh *et al.*, 2002; Nishizaki *et al.*, 2011), and morning glory (Habu *et al.*, 1998; Inagaki *et al.*, 1994). Besides transposable elements, RNA interference (RNAi) and DNA methylation are involved in unstable pigmentation in plants. For example, silencing of the *CHS* gene results in flower colour variegation in petunia (Koseki *et al.*, 2005). High methylation levels in the promoter region of *MYB10* inhibit gene expression, which causes variable colour patterns in the peel of apple (Telias *et al.*, 2011).

Peach [*Prunus persica* L. (Batsch)] is one of the most popular fruit trees in the world. Peach belongs to the Rosaceae family and serves as a model species of woody perennial angiosperms due to its small genome size of about 230Mb/haploid (The International Peach Genome Initiative, 2013). Peach trees are primarily grown for their fruit, but some cultivars are selected for their ornamental value. In China, the cultivation of ornamental peach has a long history. The ancient book Luoyanghuamuji written by Shihou Zhou in 1082 records ornamental peach cultivars such as Ersetao (variegated flower) and Ziyetao (red-leaved); and the variegated flower was highly praised in an ancient poem entitled ‘Er Se Tao’ written by Yong Shao in Song Dynasty, approximately 1000 years ago. Modern varieties have been bred for flower variegation such as Hongbaihuatao (HBH), which produces red, pink, and variegated flowers. Several studies have been conducted to investigate the genetic basis for peach flower colour. Chen *et al.* (2014) found that anthocyanin pathway genes such as *CHS*, *CHI*, and *F3H* show higher level of expression in red flowers than in white flower in ornamental peach (*Prunus persica* f. *versicolor* [Sieb.] Voss). The expression of a *MYB*-like gene (*Peace*) controls the pigmentation of flowers in the flowering peach ‘Genpei’. However, the mechanism underlying variegation in peach remains unclear.

In this study, cv. HBH was selected to investigate the molecular basis for peach flower variegation. A *GST* gene—regulator involved in anthocyanin transport (*Riant*)—was found to be associated with variegation. Levels of the Riant protein were high in red flower, but barely detectable in variegated flowers. This was due to small insertions and deletions (indels) in the last exon, resulting in a frameshift mutation. Anthocyanin accumulation in the flower of cv. HBH therefore appears to be regulated at the post-transcriptional level, unlike previous reports of transcriptional regulation in flowers of other peach varieties. This study demonstrates that the gene encoding GST is critical for anthocyanin accumulation in peach, and is helpful in understanding the mechanism underling the variegation in peach flowers.

## Materials and methods

### Plant material

Peach (*P. persica*) cultivars used in this study, including HBH, ‘Mantianhong’, ‘Hongcuizhi’, and ‘Sahongtao’, are maintained at Wuhan Botanical Garden of the Chinese Academy of Sciences (Hubei Province, China). Cv. HBH has variegation in flower colouration, while the other three cultivars bear only red flowers. Young leaves and flower buds with a diameter ranging from 0.5 to 0.8cm were collected in spring. Petals and sepalswere removed from flower samples and individually put into separate aluminium bags. All samples were immediately frozen in liquid nitrogen, and then stored at −80 °C until use.

### HPLC analysis of anthocyanin in peach flower

Anthocyanin was extracted according to a previously reported protocol (Cheng *et al.*, 2014). Approximately 0.5g of tissue was ground in liquid nitrogen and then added to 25ml of extraction solution (80:20 v/v methanol/water mixture containing 1.18mM HCl). The mixture was centrifuged at 10000 *g* for 10min. An aliquot of 10ml of supernatant was collected and evaporated under vacuum at 30 °C using a rotary evaporator. The residual was resuspended in acidified water (1.18mM HCl).

Anthocyanin was dissolved in 1ml methanol, filtered through a 0.22 μm Millipore membrane, and analysed using an HPLC-ESI-MS/MS system (ThermoFisher Scientific, Pittsburgh, PA, USA). The analytical column was a ZORBAX Extend C18, 4.6×250mm, with a particle size of 5 μm (Agilent Technologies, Waldbronn, Germany). The analytical column was sequentially eluted using mobile phase A (formic acid:water, 5:95, v/v) and mobile phase B (methanol) with a flow rate of 0.8ml/min. The linear gradient of phase B was as follows: 0–2min, 5%; 2–7min, 5–15%; 7–20min, 15–20%; 20–25min, 20–27%; 25–32min, 27%; 32–41min, 27–35%; 41.01–43min, 5%. UV-visible light detector wavelength was set at 520nm.

### Analysis of gene expression using quantitative real-time PCR

Total RNA was extracted using ZP401 kit (Zoman, Beijing, China) according to the manufacturer’s instructions. Total RNA was treated with DNase I (Takara, Dalian, China) to remove any contamination of genomic DNA. Approximately 3 μg of total RNA was used for cDNA synthesis using PrimeScriptTM RT-PCR Kit (Takara, Dalian, China). An SYBR green-based real-time PCR assay was carried out in a total volume of 20 µl reaction mixture containing 10.0 µl of 2× SYBR Green I Master Mix (Takara, Dalian, China), 0.2 µM of each primer, and 100ng of template cDNA. A peach actin gene *PpGAPDH* (*ppa008812m*) was used as a constitutive control. Primer sequences of genes involved in anthocyanin biosynthesis and transport are listed in Supplementary Table S1, available at *JXB* online.

Amplification was conducted using StepOnePlus Real-Time PCR System (Applied Biosystems, Foster, CA, USA). The amplification programme consisted of an initial denaturing step at 95°C for 30 s, followed by 40 cycles of 95°C for 30 s, and 60°C for 34 s. The fluorescent product was detected at the second step of each cycle. Melt curve analysis was performed at the end of 40 cycles to ensure the proper amplification of target fragments. Fluorescence readings were consecutively collected during the melting process from 60 to 90 °C at the heating rate of 0.5°C/sec. All analyses were repeated three times using biological replicates.

### Genomic DNA blot analysis

For cv. HBH, total DNA was separately extracted from red and variegated flowers at balloon stage, while total DNA of three cultivars used as controls (Mantianhong, Hongcuizhi, and Sahongtao), was extracted from young leaves. DNA extraction was performed using a cetyltrimethylammonium bromide (CTAB) method. Approximately 5 μg of genomic DNA was digested with *Hind*III, *Spe*I, and *Xba*I, separated on a 1.0% agarose gel, and transferred onto nylon membranes (Hybond-N, Amersham, UK) using the capillary transfer method. A pair of primers (5′-CTCAGTTCCTCTCCCGTCAG-3′/5′-CCAGCCAGATAGCTGCTCTT-3′) was designed to synthesize DNA probes using cDNA from leaves of cv. HBH as a template. The probe consisted of the last 17bp in exon 1, a complete exon 2, and a partial segment of exon 3 of *Riant*. Hybridization was carried out using the DIG Easy Hyb kit (Roche Applied Science, Indianapolis, IN, USA) according to the manufacturer’s instructions. Blots were exposed to a Lumi-Film X-ray film (Hyperfilm, Amersham) at room temperature for 25min.

### Phylogenetic analysis

The amino acid sequences of genes encoding GST from different plants were used for phylogenetic analysis. Sequence alignment was performed using CLUSTAL X, and the resulting data matrix was analysed using equally weighted neighbour joining (NJ). An NJ tree was generated using MEGA (version 5.0). Bootstrap values were calculated from 1000 replicate analyses.

### 2D electrophoresis (2-DE)

The protein extraction protocol was based on the phenol method (Hurkman and Tanaka, 1986; Ahsan *et al.*, 2008). Briefly, 2g of flower petal was ground in liquid nitrogen and added to 10ml extraction buffer containing 0.5M Tris-HCl (pH 8.3), 0.1M KCl,50mM EDTA, 2% (v/v) 2-mercaptoethanol, and 0.7M sucrose. An equal volume of Tris-HCl-saturated phenol (pH 8.0) was subsequently added and mixed by vigorous vortexing for 2min followed by centrifugation at 3500*g* for 15min. After centrifugation, the top phenol phase was collected and proteins were precipitated by adding four volumes of cold methanol containing 0.1M ammonium acetate at −20 °C for 2h. The precipitated proteins were recovered by centrifugation at 3500 *g* for 10min followed by three washes with cold methanol containing 0.1M ammonium acetate. The protein pellet was dried at room temperatureuntil needed for solubilization.

Protein concentration was quantified according to the Bradford method (Bradford, 1976). A total of 1.2mg of protein was loaded into the IPG strips (pH4–7, 24cm, Bio-Rad, USA) through rehydration for 12h at room temperature. Isoelectric focusing (IEF) electrophoresis was conducted using the following procedure: 200V for 1h (step and hold); 500V for 1.5h (step and hold); 1000V for 1h (gradient), 8000V for 2h (gradient), and 8000V for 6h (step and hold) for a total of 42000 volt-hoursusing a Protean IEF Cell (Bio-Rad). Then, the IPG strips were equilibrated for 15min in the equilibration buffer (50mM Tris-HCl pH 6.8, 6M urea, 10% v/v glycerol, 2.5% w/v SDS, and 5% 2-mercaptoethanol) containing 0.5% DTT, followed by 15min in the equilibration buffer containing 25mg/ml iodoacetamide. The second dimensional electrophoresis was run on 12% SDS-PAGE and conducted using the following procedure: 100V, 30min; 250V, 5h. The 2-DE gels were stained with Coomassie Brilliant Blue (CBB R-250).

### Trypsin digestion and MALDI-TOF-MS analysis

The differentially expressed protein spots were excised from the gel manually and washed with double-distilled water twice. The gel slices were destained, dehydrated, and digested with trypsin. The digested protein peptides were analysed over a mass range of 800–4,000Da using an Autoflex speed™ MALDI-TOF-TOF mass spectrometer (Bruker Daltonics, Bremen, Germany). Subsequently, the obtained PMF data were searched against the NCBI nr database and Swiss-Port database using MASCOT software (Mascot Wizard 1.2.0, Matrix Science Ltd., www.matrixscience.com). The parameters were set as follows: carbamidomethylation of cysteine and oxidation of methionine; peptide charge state of +1 and peptide mass tolerance of 0.5Da; a maximum of one for missed cleavages and monoisotopic.

### PAGE

PCR products were mixed with an equal volume of formamide loading buffer (98% formamide, 10mM EDTA pH 8.0, 0.025% Bromophenol Blue and Xylene Cyanol). The mixture was denatured at 94°C for 3min, and then immediately chilled on ice. An aliquot of 2 μl mixture was loaded on a 6% polyacrylamide gel, and electrophoresed for 1.5h at 1200V. Bands were visualized after silver staining, and recorded on a ScanMaker 3830 (Microtek, Shanghai, China).

### Expression vector construction and plant transformation

A pair of primers, 5′-GAATTCATGGTTGTGAAAGTGTAT GGTCC-3′/5′-CTCGAGTGGGGGTATCTCATATCTAGTAGTC-3′, was designed to amplify the whole coding region of the *Riant* gene using cDNA synthesized from flowers of cv. HBH as templates. The forward and reverse primers contained *EcoR*I and *Xho*I sites at the 5′ end, respectively. The PCR product was digested with *EcoR*I and *Xho*I, and inserted into *EcoR*I/*Xho*I-digested pSAK277 (Hellens *et al.*, 2005). The *Arabidopsis tt19* mutant (CS60000) with the Columbia genetic background was obtained from the Arabidopsis Biological Resource Center (Ohio State University, OH, USA). *Arabidopsis* transformation was performed according to the floral dip method (Clough and Bent, 1998). For transgenic plant selection, T_0_ seeds were sterilized and germinated on Murashige and Skoog (MS) medium containing 12 μg ml^−1^ kanamycin and 3% (w/v) Suc. Following 1 week of selection, kanamycin-resistant plants with red hypocotyls were transplanted to soil and placed in a growth chamber at 25°C and 50–80% relative humidity.

## Results

### Colouration and anthocyanin composition in red, pink, and variegated petals of cv. HBH

**Fig. 1.**
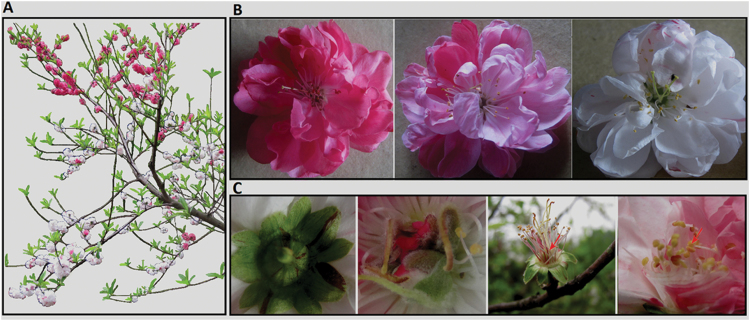
Flower colouration of ornamental cv.HBH.(A) Peach tree cv. HBH bears pigmented and variegated flowers at bloom stage. (B) Three kinds of coloured flowers within a single tree.(C) The colouration in sepal, pistil, and stamen. The arrow indicates the stamen. (A colour version of this figure is available at *JXB* online.)

Flowering peach cv. HBH produces red, pink, and variegated flowers on a single tree (Fig. 1A). The variegated flowers show a great variation in petal colouration (Fig. 1B), and can be classified into four types: white and red/pink spotted, white and pink somatic sectors, white and red somatic sectors, and pink and red somatic sectors. Most variegated flowers belong to type 1, while the other three types of variegated flowers are occasionally produced. Besides the petal tissue, variegation also appears in the colouration of sepal, stamen, and pistil tissues (Fig. 1C). Based on the flower colouration, the branches of cv. HBH can be grouped into types: red-, pink-, and white-flower branches. Red-flower branches produce exclusively red flowers with no variegated flowers. Pink-flower branches bear predominantly pink flowers, and occasionally produce pink flowers with red somatic sectors. White-flower branches bear predominantly white flowers with red/pink spotted, and occasionally produce white flowers with pink or red somatic sectors, pink flowers, and red flowers. Since the white-flower branch is the principal branch in cv. HBH, the pink- and red-flower branches are deemed to arise from bud sports.

HPLC analysis was conducted to investigate differences in anthocyanin content among red petal, pink petal, and white petal with red/pink spotted (termed variegated petalhereinafter). The red petal consists of six peaks (Supplementary Fig. S1, available at *JXB* online). Based the authors’ previous study (Cheng *et al.* 2014), peaks 1–6 correspond to cyanidin 3-galactoside, cyanidin 3-glucoside, cyanidin 3-rutinoside, peonidin 3-glucoside, cyanidin 3-rhamnoside, and peonidin 3-rutinoside, respectively. The main component in peach flower is cyanidin 3-glucoside, and its content varies greatly among red, pink, and variegated petals. The content of cyanidin 3-glucoside in red and pink petals is 12.1 and 1.26 μg/100g fresh weight (FW), respectively. However, cyanidin 3-glucoside is almost undetectable in variegated petals. In addition, the flavonol content was also determined. Overall, there is no striking difference in flavonol content among red, pink, and variegated petals. The amount of flavonol was slightly higher in variegated petals (4.8 μg/100g FW) than in red petals (4.1 μg/100g FW) and pink petals (4.6 μg/100g FW). Taken together, these results show that anthocyanin is responsible for the red and pink pigmentation in petals.

**Fig. 2.**
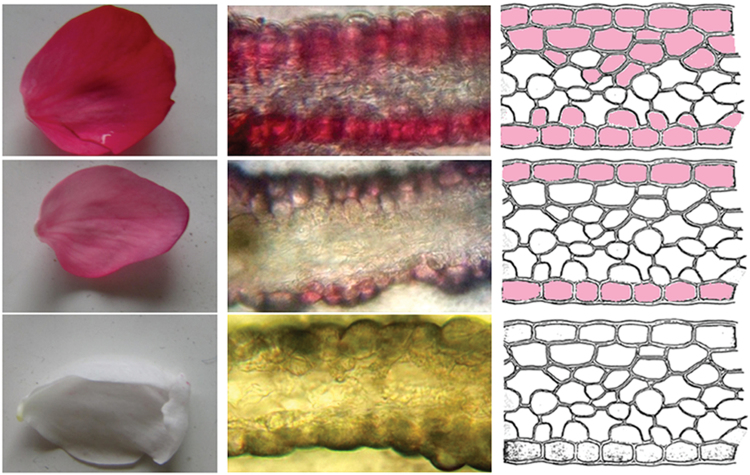
Examination of anthocyanin accumulation in petal cell layers. The middle column represents cross-sections of red, pink, and white petals (photo taken with microscope), while the right column is a diagrammatic representation of the cross-sections of red, pink, and white petals. (A colour version of this figure is available at *JXB* online.)

Microscopic analysis of fresh hand-cut sections of flower petals showed that red petals had several layers of coloured cells, with anthocyanin accumulation in both epidermal and sub-epidermal layers (Fig. 2). Pink petals only accumulated anthocyanins in the upper and lower epidermal layers. However, white petals accumulated no anthocyanins in either epidermal or sub-epidermal layers.

### Expression profiling of genes involved in anthocyanin biosynthesis and transport

**Fig. 3.**
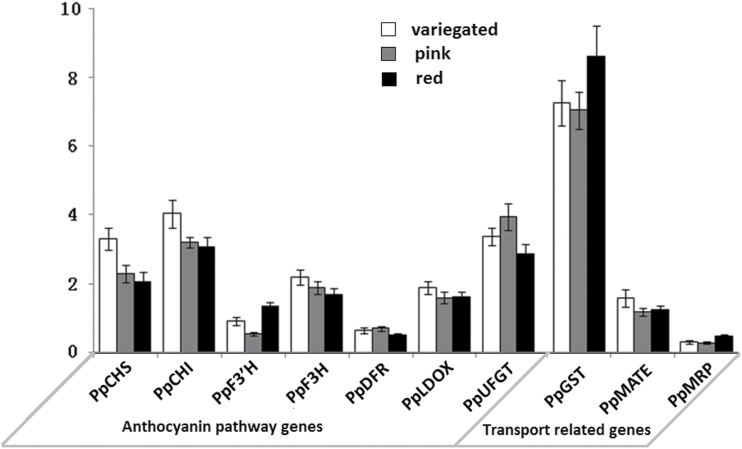
Expression level of genes involved in biosynthesis and transport of anthocyanin in petals of cv. HBH.

Red, pink, and variegated petals of flowers at balloon stage were collected to investigate the expression profile of anthocyanin biosynthesis genes using real-time PCR, including *PpCHS*, *PpCHI*, *PpF3′H*, *PpF3H*, *PpDFR*, *PpLDOX*, and *PpUFGT*. No striking difference in expression level was observed for any of these anthocyanin biosynthesis genes when comparing red, pink, and variegated petals (Fig. 3). Surprisingly, two genes—*PpCHS* and *PpCHI*—showed a significantly higher level of expression in variegated petals than in red and pink petals (*P*<0.05). Similarly, all the anthocyanin regulatory genes of *MYBs* also showed no significant difference in expression levels among pink, red, and white petals (Supplementary Fig. S2, available at *JXB* online).

Subsequently, the expression level of three genes involved in anthocyanin transport, e.g. *PpMRP*, *PpMATE*, and *PpGST*, was also measured. All these anthocyanin transport-related genes, like the anthocyanin biosynthesis genes, showed no significant difference in expression level among red, pink, and variegated petals (Fig. 3). These results suggested that anthocyanin accumulation is not regulated at the transcriptional level in the flower of cv. HBH.

### Proteomic analysis reveals a candidate GST correlated with flower colour variegation in cv. HBH

To determinate whether anthocyanin accumulation in the flower of cv. HBH is controlled at the post-transcriptional level, 2-DE protein analysis was performed. Petals from red and variegated flowers at balloon stage were chosen for 2-DE analysis. As a result, a total of 84 protein spots displaying differential abundance (>1.6-fold change) were identified (Supplementary Fig. S3). Of these proteins, 40 and 44 were up- and down-regulated in red petals, respectively.

**Fig. 4.**
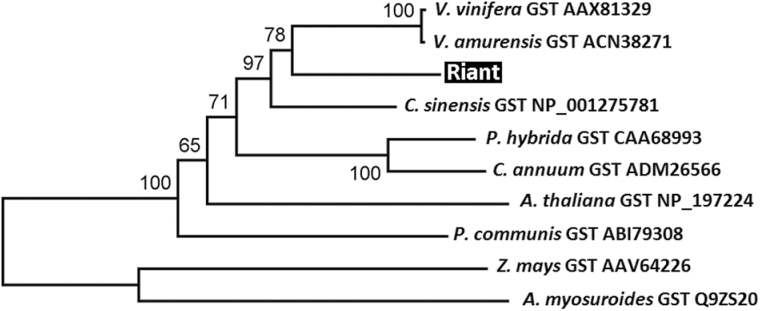
A phylogenetic tree derived from amino acid sequences of GST genes from plants. GenBank accession numbers are listed after the gene name. The *Riant* gene isolated in this study is highlighted. The numbers indicate bootstrap values calculated from 1000 replicate analyses.

These differentially abundant proteins were digested with trypsin and analysed using MALDI TOF/TOF MS/MS. Of the 84 proteins surveyed, 53 were successfully identified. Among these identified proteins (Table 1), two, PpCHS-like and PpGST with spot numbers B41 and B54, respectively, are potentially related to anthocyanin pigmentation. The PpCHS-like protein was highly expressed in red petals but undetectable in variegated petals. Coding sequences of the *PpCHS*-like gene were cloned from both red and variegated petals, but no difference was identified. Phylogenetic analysis indicated that the *PpCHS*-like gene is closely related to genes encoding a biphenyl synthase in *Malus* (Supplementary Fig. S4, available at *JXB* online). These results suggest that the *PpCHS*-like gene is unlikely involved in regulation of anthocyanin pigmentation. In contrast, the PpGST protein was found in both red and variegated petals, but its level was much higher in red petals than in variegated petals (Supplementary Fig. S4). Phylogenetic analysis showed that the *PpGST* gene is closely related to *VvGST* (Fig. 4). Since the *VvGST* gene is known for anthocyanin transport in grapevine (Gomez *et al.*, 2011), the *PpGST* gene, *Riant*, is a strong candidate responsible for variegation of flower colouration.

**Table 1.** *Proteins differentially expressed in red and white flower from* P. persica cv. HBH*

No.	Spot no.	Protein name	MW(Da)	p*I*	PSC(%)
1	A27	Geranylgeranyl pyrophosphate synthase family protein (*Populus trichocarpa*)	37305	5.06	23%
2	A42	Coatomer subunit epsilon-2-like (*Fragaria vesca* subsp. vesca)	32395	5.16	9%
3	A49	Triose phosphate isomerase cytosolic isoform-like protein (*Capsicum annuum*)	27433	6.00	37%
4	A68	PREDICTED: peroxiredoxin-2E, chloroplastic-like (*F. vesca*)	24230	8.96	20%
5	A48	S-locus lectin protein kinase family protein (*Theobroma cacao*)	87288	6.64	1%
6	A22	Calcium-binding EF hand family protein (*T. cacao*)	31298	4.8	18%
7	A62	Adenine nucleotide hydrolases-like superfamily protein (*T. cacao*)	17992	6.2	48%
8	A64	Hypothetical protein	17685	4.77	14%
9	A33	PREDICTED: 14-3-3-like protein-like (*F. vesca* subsp. vesca)	29687	4.77	29%
10	A55	Chalcone-flavanone isomerase family protein (*T. cacao*)	32276	7.77	35%
11	A26	Fructose-bisphosphate aldolase 4 (*Camellia oleifera*)	42688	8.15	16%
12	A38	EF hand family protein, expressed isoform 1 (*T. cacao*)	30276	6.44	13%
13	A28	Temperature-induced lipocalin (*P. persica*)	21450	5.60	38%
14	A44	ATP-dependent Clp protease proteolytic subunit 4 (*T. cacao*)	32028	5.97	19%
15	A13	Actin 7 (*Arabidopsis thaliana*)	41954	5.31	50%
16	A58	Cyclophilin peptidyl-prolyl *cis-trans* isomerase family (*T. cacao*)	15270	5.61	45%
17	A23	Annexin-like protein RJ4 (*P. trichocarpa*)	35923	6.19	49%
18	A24	RNA-binding protein Nova-1-like (*Vitis vinifera*)	30618	6.01	38%
19	B15	Full=UDP-sugar pyrophosphorylase	67671	5.71	21%
20	B29	26S proteasome non-ATPase regulatory subunit 4-like (*F. vesca*)	43042	4.48	19%
21	B35	Monodehydroascorbate reductase (*Malus domestica*)	47111	6.51	26%
22	B40	Isovaleryl-CoA dehydrogenase 1, mitochondrial-like (*F. vesca*)	43644	6.20	23%
23	B41	Chalcone synthase 1-like (*F. vesca* subsp. vesca)	43390	5.97	32%
**24**	**B54**	**GST-like protein (*M. domestica***)	**24389**	**5.34**	**37%**
25	B28	TCP domain class transcription factor (*M. domestica*)	57477	5.72	40%
26	B23	RNA-binding KH domain-containing protein isoform 1 (*T. cacao*)	59072	6.12	35%
27	B09	Lipoxygenase (*M. domestica*)	90278	5.40	20%
28	B21	Starch synthase isoform I (*Manihot esculenta*)	71556	5.38	22%
29	B48	Papain family cysteine protease (*T. cacao*)	40868	5.86	17%
30	B17	Mediator of RNA polymerase II transcription subunit 37e-like (*F. vesca*)	69534	5.25	20%
31	B03	Patellin-3-like (*F. vesca* subsp. vesca)	65404	4.85	6%
32	B01	Heat shock protein 70 (Hsp 70) family protein isoform 1 (*T. cacao*)	100326	5.40	20%
33	B27	TCP domain class transcription factor (*M. domestica*)	57477	5.72	44%
34	B05	Patellin-3-like (*F. vesca* subsp. vesca)	65404	4.85	10%
35	B16	NADP-malic protein (*Prunus armeniaca*)	65358	5.73	24%
36	B33	3-Ketoacyl-acyl carrier protein synthase I (*T. cacao*)	52446	6.38	23%
37	B19	DC1 domain-containing protein (*T. cacao*)	65260	4.80	31%
38	B06	Cytosolic aconitase (*Pyrus pyrifolia*)	108637	6.98	10%
39	B58	PREDICTED: allene oxide cyclase 4, chloroplastic-like (*F. vesca*)	20527	5.41	19%
40	B55	Chaperonin 20 isoform 1 (*T. cacao*)	26399	7.79	11%
41	B44	Caffeic acid 3-*O*-methyltransferase 1 (*T. cacao*)	42020	5.40	21%
42	B25	TCP domain class transcription factor (*M. domestica*)	45351	5.26	26%
43	B18	UDP-sugar pyrophosphorylase (*T. cacao*)	67671	5.71	14%
44	B31	Hypothetical protein	39546	6.77	7%
45	B20	Nucleoporin nup211-like (*Glycine max*)	54774	5.99	16%
46	B24	Glucose-6-phosphate 1-dehydrogenase	59358	6.13	24%
47	B50	Prolyl 4-hydroxylase alpha subunit, putative (*Ricinus communis*)	33559	6.26	36%
48	B13	Oligopeptidase A-like (*F. vesca* subsp. vesca)	90582	6.42	12%
49	B49	Probable rhamnose biosynthetic enzyme 1-like (*Citrus sinensis*)	33636	6.17	21%
50	B57	Peroxiredoxin-2B-like (*F. vesca* subsp. vesca)	17480	5.70	57%
51	B60	Regulator of ribonuclease-like protein 2-like (*F. vesca* subsp. vesca)	18008	5.69	64%
52	B51	Protein PPLZ12, putative (*R. communis*)	31730	5.27	41%
53	B65	Ribonuclease UK114-like (*F. vesca* subsp. vesca)	19977	8.99	44%

* The protein associated with the variegated colouration of the peach flower is highlighted in bold.

### Small indels in the *Riant* gene and their association with variegation in petal colouration

**Fig. 5.**
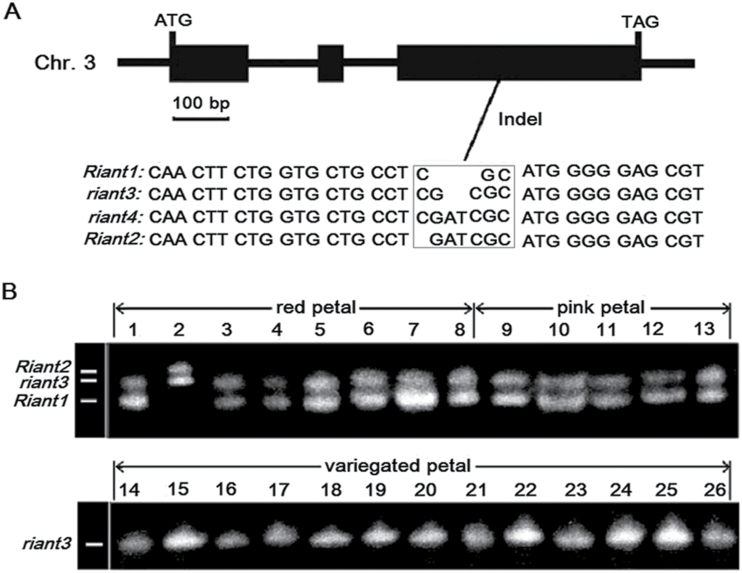
The *Riant* gene isolated from peach cv. HBH. (A) Genomic structure and genetic variation highlighted in a square box. (B) Genotyping of red, pink, and variegated flowers based on the indel in the last exon of the *Riant* gene; the detected alleles are indicated.

To confirm that the *Riant* gene is responsible for petal variegation, its coding sequences from red, pink, and variegated petals were cloned and sequenced. Comparison of the coding sequences revealed a 2-bp insertion in the third exon of *Riant* (Fig. 5A). The 2-bp insertion causes a frameshift and a premature stop codon. Subsequently, a pair of primers flanking the 2-bp insertion, 5′-CTCTGGTGGATCAGTGGCT-3′ (GIF) and 5′-TATCCCTGGAAGATGGCTC-3′ (GIR), was designed to amplify red, pink, and variegated petals from different clones of cv. HBH (Fig. 5B). Interestingly, all the red or pink petals contained two bands, suggesting they are heterozygous at the *Riant* locus. Three alleles, designated *Riant1*, *Riant2*, and *riant3*, were detected among the red and pink petals. Of the 13 pigmented petals tested, 12 have a *Riant1*/*riant3* genotype. One (the second sample in Fig. 5B) has a *Riant2*/*riant3* genotype. Sequencing of the PCR products revealed that both *Riant1* and *Riant2* have an intact ORF, while the *riant3* allele has a frameshift mutation due to a 2-bp insertion in the third exon (Fig. 5A). It is worth noting that *Riant2* contains a 3-bp insertion in the third exon, so should not induce a frame shift. All the variegated petals amplified only one band that corresponds to the *riant3* allele, suggesting they are homozygous at the *Riant* locus.

To determine whether there are other alleles of the *Riant* gene, 15 more flowers (five with red petals, five with pink petals, and five with variegated petals) were randomly collected from different clones of cv. HBH. The petals of these flowers were individually subjected to genomic DNA extraction, and the extracted DNA was subsequently amplified using the primers GIF/GIR as mentioned above. Cloning and sequencing of the PCR products revealed one more frameshift mutant allele from the variegated flower, designated *riant4*, which contains a 4-bp insertion (Fig. 5A). All four alleles—*Riant1* to *Riant4*—were deposited in GenBank under accession nos. KT312847 to KT312850, respectively.

### Copy number of the *Riant* gene in the peach genome

**Fig. 6.**
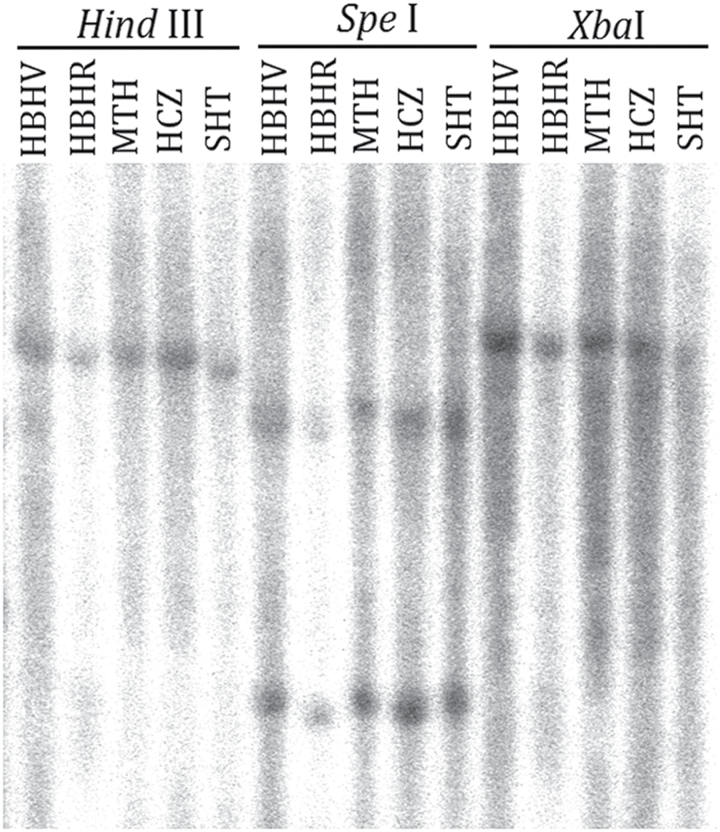
Southern blot analysis of peach genomic DNA. The *Riant*-specific probe consists of a partial sequence of the first exon, whole fragment of the second exon, and a partial sequence of the third exon that covers the indel site. HBHV and HBHR represent variegated and red flowers from cv. HBH, respectively. MTH, Mantianhong; HCZ, Hongcuizhi; SHT, Sahongtao.

In many species, variegation in flower colouration is caused by transposon activity. To determine whether the small indels in the third exon of the *Riant* gene have also arisen from a transposon or other DNA fragment that was not amplified by the primers GIF and GIR, aDNA gel blot analysis was performed. Three cultivars (Mantianhong, Hongcuizhi, and Sahongtao) that bear red flowers were used as controls. Genomic DNA was digested with *Spe*I, *Hind*III, and *Xba*I. The digested DNA was hybridized with a probe covering the indel site in the third exon of the *Riant* gene. Both *Hind*III and *Xba*I digestions yielded a single hybridizing DNA band in all the tested samples, while there were two hybridizing bands for *Spe*I digestion in all the tested samples (Fig. 6). This result indicates that only one copy of the *Riant* gene is present in the peach genome, and it is not interrupted by a transposon. In other words, the small indel mutation in the *Riant* gene is not due to insertion and excision of a transposable element.

### Functional analysis of the *Riant* gene in the *Arabidopsis* transparent testa19 mutant

**Fig. 7.**
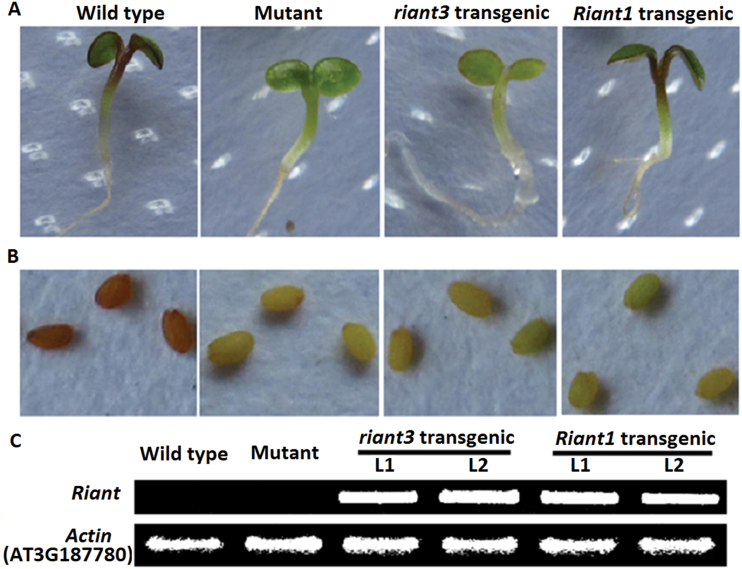
Complementation of the pigmentation of *Arabidopsis tt19* mutant seedlings of the ecotype Columbia with the *Riant* gene.(A) Phenotypes of wild-type, mutant, and transgenic *Arabidopsis* seedlings. (B) Phenotypes of wild-type, mutant, and transgenic *Arabidopsis* seeds. (C)Expression level of the *Riant* gene in wild-type, mutant, and transgenic *Arabidopsis* seedlings. Two transgenic lines each of *Riant1* and *riant3* were analysed, and these exhibited similar phenotypes, as shown. (A colour version of this figure is available at *JXB* online.)

The *Arabidopsis* transparent testa19 (*tt19*) mutant, lacking GST, was selected to investigate the functionality of the *Riant* gene. The coding sequences of both *Riant1* and *riant3* alleles were separately transferred into the *Arabidopsis tt19* mutant under the control of the cauliflower mosaic virus 35S promoter, and several transgenic lines were generated for each construct. Seeds of the *Arabidopsis tt19* mutant, T1 transgenic lines, and wild-type *Arabidopsis* were germinated and grown on MS medium. Germinating seedlings of wild-type plants and transgenic lines expressing *Riant1* had red hypocotyls, whereas hypocotyls of the *Arabidopsis tt19* mutant and transgenic lines expressing *riant3* were green (Fig. 7A). Moreover, seeds collected from kanamycin-resistant T1 plants and the *Arabidopsis tt19* mutant were pale brown in colour, while seeds of wild-type plants were dark brown in colour (Fig. 7B). This suggests that Riant, like petunia AN9, complements the anthocyanin accumulation in vegetative tissues, but not the brown pigmentation in the seed coat (Kitamura *et al.*, 2004). In addition, reverse transcription (RT)-PCR analysis showed that both *Riant1* and *riant3* were highly expressed in transgenic lines (Fig. 7C). Taken together, these results demonstrated that, the *Riant1* allele is involved in the transport of anthocyanins from cytosol to vacuole, but the *riant3* allele is nonfunctional.

## Discussion

### The *Riant* gene is involved in anthocyanin transport and is critical for flower colouration in peach cv. HBH

In plants, flower pigmentation is mainly attributed to anthocyanin accumulation (Bogs *et al.*, 2005; Cutanda-Perez *et al.*, 2009). In this study, HPLC analysis revealed that cyanidin 3-glucoside is the main component of anthocyanin in flowers of cv. HBH, which is consistent with previous reports (Chaparro *et al.*, 1995*a*; Cheng *et al.*, 2014; Uematsu *et al.*, 2014). Both red and pink petals accumulate cyanidin 3-glucoside, and its content is approximately 10-fold higher in red petals than in pink petals. In contrast, cyanidin 3-glucoside is almost undetectable in white sectors of the variegated petal. This suggests that anthocyanin accumulation contributes to flower colouration in cv. HBH and its flower colour variegation is related to a change in anthocyanin accumulation.

In contrast to anthocyanin, flavonol shows the highest level of accumulation in the variegated petal, followed by pink and red petals. This is consistent with previous findings that blocking anthocyanin accumulation strengthens the metabolic flux towards flavonols (Gou *et al.*, 2011; Sun *et al.*, 2012). Real-time PCR analysis reveals that early biosynthetic genes such as *CHS*, *CHI*, and *F3H* show higher levels of expression in variegated petals than in red and pink petals, whereas, the expression levels of late biosynthetic genes such as *DFR*, *LDOX*, and *UFGT* are not significantly different among red, pink, and variegated petals. Thus, it seems that anthocyanin and flavonol biosynthesis is coordinately regulated by anthocyanin pathway genes (Owens *et al.*, 2008; Han *et al.*, 2010). However, all the potential *MYB* regulatory genes tested in this study showed no significant difference in expression level among red, pink, and white petals of cv. HBH.

The vacuole is the cellular compartment where anthocyanins accumulate. Increasing evidence shows that GST is indispensable for the transport of anthocyanins from the ER to the vacuole (Marrs *et al.*, 1995; Alfenito *et al.*, 1998; Zhao and Dixon, 2010; Gomez *et al.*, 2011; Gou *et al.*, 2011). This study also demonstrates that the *Riant* gene encoding GST is essential for anthocyanin pigmentation in peach cv. HBH. In variegated petals, two alleles of the *Riant* gene were identified, but both of them encode truncated nonfunctional proteins due to 2- or 4-bp insertions in the third exon that result in frameshift mutations. Interestingly, the mutation of the *Riant* gene does not alter the expression level of genes involved in anthocyanin biosynthesis. Similar results have been reported in the *Arabidopsis tt19* mutant (Sun *et al.*, 2012). In *Arabidopsis*, anthocyanin accumulates first in vesicles and then is transported to the vacuole via fusion with the tonoplast (Poustka *et al.*, 2007). Knockout of the *GST* gene results in weak accumulation of anthocyanins in vesicles, but not in the vacuole (Goodman *et al.*, 2004; Gomez *et al.*, 2011; Li *et al.*, 2011; Sun *et al.*, 2012). In peach,no anthocyanin was foundin white flowers, but some was present in variegated petals. Anthocyanins may be temporarily accumulated in vesicles, but subsequently degraded in variegated flowers of cv. HBH. In addition, the *PpMATE* gene shows a higher level of expression in variegated petals than in red and pink petals. This suggests a relationship between the *PpMATE* and *Riant* genes, to coordinately transport anthocyanins from the ER to the vacuole. MATE is involved in anthocyanin transport via the transporter-mediated mechanism, suggesting that both vesicle-mediated trafficking and MATE transporter-mediated mechanisms are involved in the sequestration of anthocyanins to vacuoles in peach.

Genetic mapping reveals that two loci, *B* and *Fc*, are responsible for flower colour in peach. The *B* locus has been mapped to an interval flanked by two markers, Pr1-12 and BPPCT028, on the bottom of chromosome 1 (Martínez-Gómez *et al.*, 2007), while the *Fc* locus is anchored to an interval flanked by two markers, OPJ01 and MA039a, on the upper region of chromosome 3 (Yamamoto *et al.*, 2005). The *Peace* gene that regulates petal pigmentation in peach ‘Genpei’ is located at the bottom of chromosome 1 (Uematsu *et al.*, 2014). The peach reference genome (The International Peach Genome Initiative, 2013) has been searched, and the *Riant* gene was found to be located at the top of chromosome 3. Thus, it is worthy of further study to clarify whether the *Peace* and *Riant* genes are actually candidates of the *B* and *Fc* loci, respectively.

In *Arabidopsis*, TT19 participates in both anthocyanin accumulation in vegetative tissues and proanthocyanidin (PA) accumulation in seed coats (Kitamura *et al.*, 2004). The accumulation of PA pigments is responsible for brown colouration in *Arabidopsis* seed coats. Petunia anthocyanin 9 (AN9) is an orthologue of *Arabidopsis* TT19. However, its ectopic expression in the *Arabidopsis tt19* mutant complements the anthocyanin accumulation in vegetative tissues, but not the brown pigmentation in the seed coat (Kitamura *et al.*, 2004). Like *AN9*, *Riant* also complements the anthocyanin phenotype, but not the PA defect of the *tt19* mutant.

### Potential mechanism underlying the mutation of the *Riant* gene in flowering peach cv. HBH

Transposable elements are often responsible for the phenotype of colour variegation in plants (Habu *et al.*, 1998; van Houwelingen *et al.*, 1998; Pooma *et al.*, 2002; Schwinn *et al.*, 2006; Nishizaki *et al.*, 2011; Lazarow *et al.*, 2012). In this study, variegation in peach was shown to be associated with small indels in the last exon of the *Riant* gene. Both red and pink petals are heterozygous at the *Riant* locus, with a functional allele and a frameshift mutant allele, whereas variegated petals contain two nonfunctional alleles (homozygous, Fig. 5). Moreover, DNA blot analysis shows that there is no polymorphism between red and variegated petals. These results strongly suggest that there are no transposable elements in the *Riant* locus in cv. HBH.

Flowering peach cv. HBH is quite similar in variegation to the previously reported peach cultivar Pillar, which bears dark pink, light pink, and white flowers on the same tree (Chaparro *et al.*, 1995*a*). The phenotype in cv. Pillar is assumed to be controlled by an active transposable element in the *W* locus (Chaparro *et al.*, 1995*b*). If the dark pink flowers carry a functional *W* allele that is reverted by excision of the transposable element, its self-pollinated progeny are expected to segregate for flower colouration. However, self-pollinated seeds of the dark pink flowers on Pillar trees yield only anthocyanin-deficient progeny (Chaparro *et al.*, 1995*b*). This suggests that the unstable phenotype in cv. Pillar cannot be ascribed to an active transposable element. Similarly, no transposable element is identified in the *Peace* gene responsible for the variegated phenotype in flowering peach ‘Genpei’ that produces pink and variegated flowers on the same tree (Uematsu *et al.*, 2014). Thus, it appears that flower colour variegation is not due to transposable elements. DNA methylation and RNAi can also cause colour variegation in plants (Koseki *et al.*, 2005; Telias *et al.*, 2011). These two mechanisms lead to down-regulation of the anthocyanin pathway. However, expression levels of genes were similar among red, pink, and variegated flowers. This suggests colour variegation in cv. HBH is controlled at the translational level, and is unlikely to be related to DNA methylation or RNAi.

A total of four alleles were identified at the *Riant* locus. Of these alleles, *Riant1* has the same coding sequence as the *GST* gene (*ppa011307m*) retrieved from the peach reference genome of cv. Lovell (The International Peach Genome Initiative 2013). All the tested flowers contain the *riant3* allele. Thus, the *riant3* allele probably represents an original allele in cv. HBH, whereas, *Riant1*, *Riant2*, and *riant4* are mutants of the *riant3* allele. These small indels appear responsible for the DNA variation at the *Riant* locus.

Strand slippage during DNA replication is a well-understood mechanism of the small indel mutagenesis (Garcia-Diaz and Kunkel 2006; Montgomery *et al.* 2013), and the G–C pairing surrounding the indels contributes to the rate of small indels (Tanay and Siggia, 2008). The *Riant1* allele has a 2-bp (GC) deletion compared with the *riant3* allele. Interestingly, the indel polymorphic locus of the *riant3* allele contains a (GC)2 sequence, which has the potential to induce strand slippage. Thus, strand slippage may be responsible for the DNA variation at the *Riant* locus. Besides strand slippage, DNA single- or double-stranded breaks are also required for the generation of small indels as specialized translesion synthesis polymerases are capable of bypassing DNA lesions without repairing them, which allows replication on damaged DNA substrates and, in some cases, to promote mutagenic DNA synthesis (Waters *et al.*, 2009; De and Babu, 2010; Roerink *et al.*, 2012). For example, *Sulfolobus solfataricus* DNA polymerase IV (Dpo4), a member of the Y family of DNA polymerases, can generate deletions and mismatches at an unusually high average rate and preferentially at cytosine flanked by 5′-template guanine (Goodman, 2002). In this study, small indels also occur at the site with GC nucleotide sequences. Therefore, it cannot be excluded that the DNA variation of the *Riant* gene may be caused by similar mechanisms.

### Relationship between somatic chimerism and flower colour variegation in cv. HBH

Chimerism is one of the factors that causes variegated colouration in plants (Stewart and Dermen, 1979; Mandal *et al.*, 2000; Walker *et al.*, 2006; Pelsy, 2010). Dicotyledonous plants usually have stratified apical meristems containing three layers of dividing cells, L1, L2, and L3, and each layer contributes to different tissues of the developing organs (Carles and Fletcher, 2003). The word chimera indicates that one or more layers consist of genetically distinct cells, and chimerascan be classified as periclinal, mericlinal, or sectorial chimeras. For floral meristems, layers L1 and L2 contribute to epidermis and internal tissues, respectively, while layer L3 forms the innermost tissues such as vascular tissues (Brand *et al.*, 2001; Filippis *et al.*, 2013). Thus, layers L1 and L2 play an important role in determining flower colouration (Stewart and Dermen, 1979; Carles and Fletcher, 2003).

**Fig. 8.**
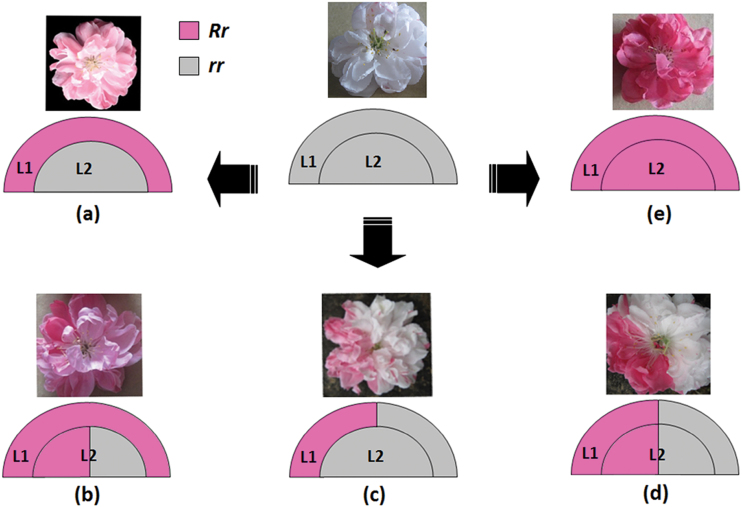
A proposed model for the variegated phenotype in flower colouration of peach cv. HBH. L1 and L2 indicate different layers of floral meristems, and *R* and *r* represent functional and nonfunctional alleles of the *Riant* gene, respectively. White flower with red/pink spots carrying two nonfunctional alleles of the *Riant* gene. (a) Pink flower derived from periclinal chimera, (b) pink flower with red somatic sectors derived from mericlinal chimera, (c) white flower with pink somatic sectors derived from mericlinal chimera, (d) white flower with red somatic sectors derived from sectorial chimera, (e) red flower carrying one functional and one nonfunctional allele of the *Riant* gene. (A colour version of this figure is available at *JXB* online.)

Anthocyanin content in pink-coloured petals is extremely low—only approximately 10% of that in red-coloured petals. A similar result is also observed in grapevine. ‘Cabernet Sauvignon’ and its bud sport ‘Malian’ bears dark red and pink berries, respectively, with Malian berry containing only 10% of the anthocyanin content of the Cabernet Sauvignon berry (Boss *et al.*, 1996). In Cabernet Sauvignon, both the L1 and L2 layers carry one red and one white allele, giving rise to a coloured epidermis derived from the L1 layer and several sub-epidermal coloured layers in the skin derived from L2 (Walker *et al.*, 2006). In contrast, Malian is a periclinal chimera with the L1 layer carrying one red and one white allele of the berry colour locus while two white alleles for the L2 layer, resulting in only the epidermis containing anthocyanin. The pink flower of peach cv. HBH is a periclinal chimera with the L1 layer capable of accumulating anthocyanin while the L2 layer cannot. Since both pink and red flowers belong to bud sports and are heterozygous for the *Riant* gene responsible for anthocyanin accumulation, the L1 layer in the pink flower should be heterozygous with one functional allele such as *Riant1* or *Riant2*, whereas, both the L1 and L2 layers in the red flower are heterozygous. The variegated flowers, including pink and red somatic sectors (type 1), pink and white somatic sectors (type 2), and red and white somatic sectors (type 3), can be attributed to the existence of genetically distinct cells within the same layer. Thus, a model is proposedfor the variegated phenotype in flower colouration of peach cv. HBH (Fig. 8).

The type 1 variegated flower results from a sectorial chimera, whereas, both the type 2 and type 3 variegated flowers have arisen from a mericlinal chimera. These three types of variegated flowers occur at low frequency, with no branches that produce predominantly one of the three types of variegated flowers. This is consistent with a previous finding that sectorial and mericlinal chimeras are unstable (Pelsy, 2010; Filippis *et al.*, 2013). In contrast, periclinal chimeras are very stable giving rise to the pink-flower branch that bears predominantly pink flowers.

No pure white flowers are found on the trees of cv. HBH, and the petals carrying no functional *Riant* allele still have red- and pink-coloured spots. This is consistent with previous reports that knockout of the *GST* gene cannot completely inhibit anthocyanin accumulation in maize (Goodman *et al.*, 2004) and *Arabidopsis* (Li *et al.*, 2011; Sun *et al.*, 2012). The development of white with red/pink spotted flowers could be explained by the following reasons. First, mutated cells capable of accumulating anthocyanin appear at late stages of floral meristem development, and they are distributed within the L1 and/or L2 layers. An invasion by cells from the inner L2 layers into the outer L1 layer, termed ‘displacement’, has been reported in grapevine (Hocquigny *et al.*, 2004; Walker *et al.*, 2006; Pelsy, 2010). Thus, these mutated cells will be mixed with the wild-type cells incapable of accumulating anthocyanin in petal tissue, resulting in white and red/pink spotted flowers. Second, functional redundancy in the *GST* gene family has been reported in grapevine (Conn *et al.*, 2008). Thus, it is unclear whether other anthocyanin carrier(s) could complement anthocyanin accumulation, resulting in variegated colouration.

This study reveals that the *Riant* gene encoding GST is essential for flower colouration in peach. Mutations involving small indels frequently occur in the last exon of the *Riant* gene, which causes the variegated flower in peach cv. HBH. However, the mechanism underlying the small indel formation requires further study.

## Supplementary data

Supplementary data are available at *JXB* online.

Table S1. Primers for real-time PCR.

Fig. S1. HPLC analysis of anthocyanin composition in red, pink, and variegated flowers of cv. HBH.

Fig. S2. Expression level of potential regulatory genes involved in anthocyanin biosynthesis in petals of cv. HBH.

Fig. S3. Gel image of proteins separated by 2D PAGE.

Fig. S4. A phylogenetic tree of CHS genes from different plant species.

Supplementary Data
