# Supplementary material for: A small indel mutation in an anthocyanin transporter causes variegated colouration of peach flowers
Source: J Exp Bot. 2015 Sep 10;66(22):7227–39. doi: 10.1093/jxb/erv419 (PMC4765791; doi:10.1093/jxb/erv419)
Supplement: Supplementary Data [file supp_66_22_7227__index.html]

A small indel mutation in an anthocyanin transporter causes variegated colouration of peach flowers — A small indel mutation in an anthocyanin transporter causes variegated colouration of peach flowers — Supplementary Data 

# A small indel mutation in an anthocyanin transporter causes variegated colouration of peach flowers

## Supplementary Data

Data files

- Supplementary Data - Supplementary Data
