# Supplementary material for: A small indel mutation in an anthocyanin transporter causes variegated colouration of peach flowers
Source: J Exp Bot. 2015 Sep 10;66(22):7227–39. doi: 10.1093/jxb/erv419 (PMC4765791; doi:10.1093/jxb/erv419)
Supplement: Supplementary Data [file supp_erv419_jexbot153379_file001.pdf]

A small indel mutation in an anthocyanin transporter causes variegated coloration of peach flowers

Jun Cheng[1,2], Liao Liao[1], Hui Zhou[1,2], Chao Gu[1], Lu Wang[1], Yuepeng Han[1,*]

[1]Key Laboratory of Plant Germplasm Enhancement and Specialty Agriculture, Wuhan Botanical Garden of the Chinese Academy of Sciences, Wuhan, 430074, P.R. China

[2]Graduate University of Chinese Academy of Sciences, 19A Yuquanlu, Beijing, 100049, P.R. China

* Corresponding author: yphan@wbgcas.cn

Tel/Fax: 86-27-8751-0872

Table S1 Primers for Real time PCR

Gene name	GDR accession number	Forward primer	Reverse primer
PpCHS	ppa006888m	5'-CAGCGCATGTGTGACAAATCT-3'	5'-ATTTTGGATTTGGGCTGGC-3'
PpCHI	ppa011276m	5'-GAGATCGTTACAGGTCCATTTG-3'	5'-GTGGGAAGTTTTGATCCTTGA-3'
PpF3H	ppa007636m	5'-GGACTGGACACAGAGGCATT-3'	5'-AATTGTGCCTGGGTCAGTGT-3'
PpF3'H	ppa004433m	5'-CTCTCGCTCAAAGAGGATGC-3'	5'-CCATTCCACTGTGCTTGATG-3'
PpDFR	ppa008069m	5'-CTCCAAGTTCTTCTCCGTCAA-3'	5'-CGGCAGCTTTCGATTTCTC-3'
PpLDOX	ppa007738m	5'-AGGAGTTGAAGAAGGCAGCA-3'	5'-GCCTGGTCATTGGCATACTT-3'
PpGST	ppa011307m	5'-ACTTCAACTTCTGGTGCTGC-3'	5'-GCTGCTCTTTGATAATCTTTCC-3'
PpMATE	ppa004822m	5'-ATTGGTTTTCTGGCCCTTAT-3'	5'-ACTCCACCCTTCATTGCAC-3'
PpMRP	ppa000145m	5'-CCGAGTCCTTCTGCTTGATG-3'	5'-CAACCTGTTTTCCCCTTCC-3'
PpUFGT	ppa005162m	5'-TGGATAGGCGGACCACACTC-3'	5'-CAGGGGCTAAATCATCAAAAG-3'
PpMYB10.1	ppa026640m	5'-GAAATGATTGGTGGGAAACC-3'	5'-GTCCTTCTTCTGAAACATTGGT-3'
PpMYB10.2	ppa016711m	5'-CACCATCAACAAGGATTGGA-3'	5'-CCGATTGTGGCATATCATCA-3'
PpMYB10.3	ppa020385m	5'-AAGGCCACAACCAAGAAGA-3'	5'-AACCCAAGACCAGAACCTGT-3'
PpMYB10.4	ppa018744m	5'-AACTGCCAATACTACCCTCATC-3'	5'-TGAAGTGGTCTTCCTCTAGCA-3'
PpMYB10.5	ppa022808m	5'-GTCGTTGATTGCTGGAAGAA-3'	5'-TTTATCTTTTTCGGCCTCAGA-3'
PpMYB10.6	ppa024617m	5'-GTCGCTGATTGCTGGAAGAC-3'	5'-GAGAAGGTTCGTGGTTGAGG-3'
Peace	AB897865	5'-GTCCACAGATAAAACTCAACC-3'	5'-CCTTCTTCTTCGCCACCAA-3'
PpGAPDH	ppa008812m	5'-TGCCATTGAAATCCTGAAAC-3'	5'-ACCAATTGGATCATCCTCCT-3'

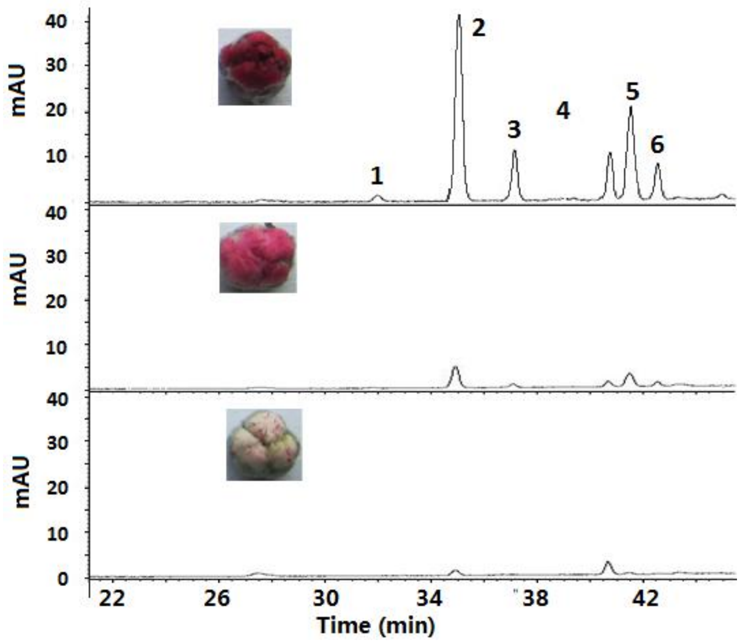

Fig. S1 HPLC analysis of anthocaynin composition in red, pink, and variegated flowers of cv. HBH. Absorbance spectrum of the wave length was set at 520 nm. 1, cyanidin 3-galactoside; 2, cyanidin 3-glucoside; 3, cyanidin 3-rutinoside; 4, peonidin 3-glucoside; 5, cyanidin 3-rhamnoside; 6 peonidin 3-rutinoside.

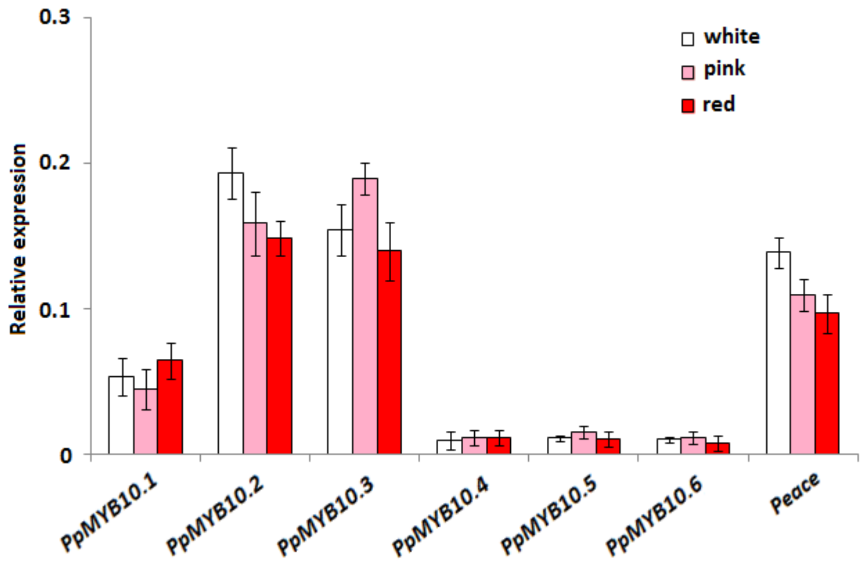

Fig. S2 Expression level of potential regulatory genes involved in anthocyanin biosynthesis in petals of cv. HBH.

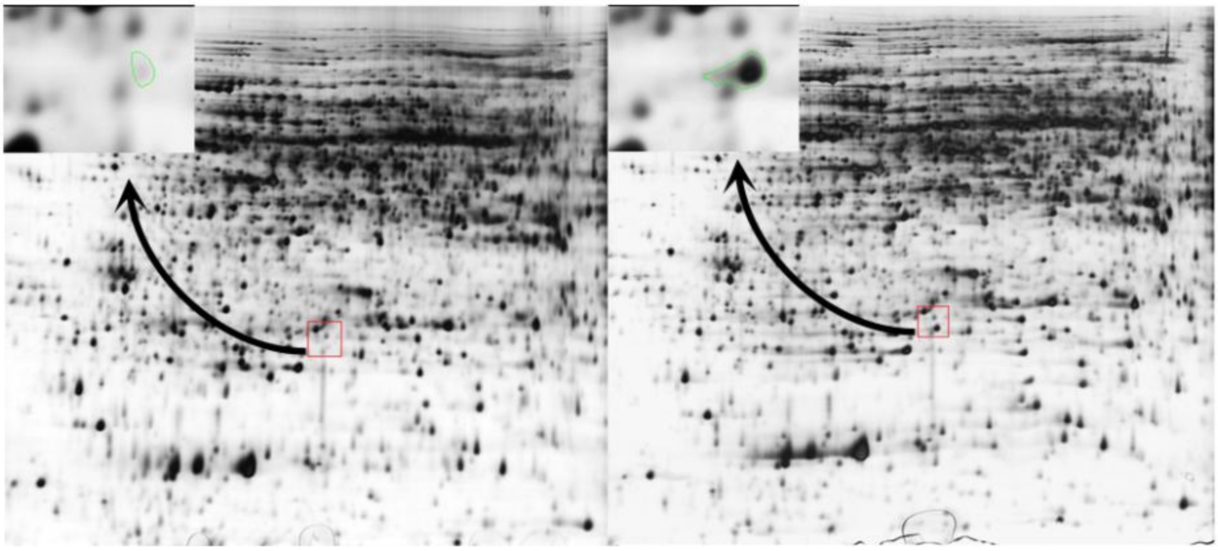

Fig. S3 Gel image of proteins separated by two-dimensional polyacrylamide gel electrophoresis (2-D PAGE). Left, variegated flower from cv. HBH; Right, red flower from cv. HBH. The differentially expressed protein PpGST is highlighted in an enlarged gel image in the top-left corner. The protein dots marked with a green circle correspond to the *Riant* gene.

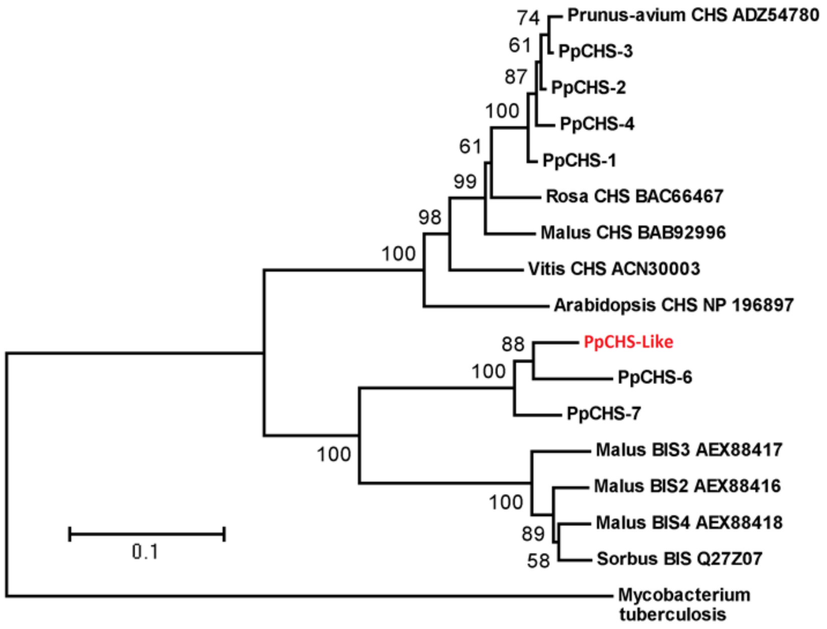

Fig. S4 A phylogenetic tree of CHS genes from different plant species. Numbers beside the nodes indicate bootstrap values calculated from 1,000 replicate analyses. The scale bar indicates the nucleotide substitution ratio. The peach *PpCHS-like* gene isolated in this study is heighted in red color.
